# Minimally Invasive Excision of Breast Masses under Ultrasound Guidance: A Single Center's Five-Year Experience on the Breast Lesion Excision System

**DOI:** 10.1155/2022/1888726

**Published:** 2022-02-04

**Authors:** Hakki Muammer Karakas, Gulsah Yildirim

**Affiliations:** University of Health Sciences, Istanbul Fatih Sultan Mehmet Training and Research Hospital, Department of Radiology, Istanbul, Turkey

## Abstract

**Background:**

The purpose of this study was to investigate the feasibility of the percutaneous radiofrequency (RF) excision system (BLES) as a primary method of diagnosis and removal of small breast masses.

**Methods:**

Ninety-six lesions in 95 patients with 50.5 ± 8.4 years of age were treated in a five-year period by a single operator. Inclusion criteria were as follows: size (<20 mm), depth (>10 mm), and indeterminate or suspicious radiological features (74 BI-RADS 3 and 22 BI-RADS 4). The procedure was performed under ultrasound (US) guidance using 6 G retriever probes with 12-, 15-, and 20-mm baskets.

**Results:**

Lesions were between 5 and 20 (12.3 ± 3.8) mm in length. They were removed at the first attempt in all but one case. The technical success rate was 98.95%, and the diagnostic success rate was 100%. Ninety-one lesions were histologically benign and five were neoplastic. Two lesions that were previously classified as BI-RADS 3 were diagnosed as neoplasia (atypical lobular hyperplasia), and nineteen lesions that were previously classified as BI-RADS 4a were diagnosed as benign. The complete excision rate (presence of tumor-free negative surgical margin) was 40% in neoplastic lesions. There were no major complications. The minor complication rate was 1.58%. No recurrence was observed during 18 months of follow-up.

**Conclusion:**

BLES delivers surgical quality specimens for confident histopathological examination and is a safe alternative to surgical resection in lesions with suitable size.

## 1. Introduction

Breast cancer is the most common cancer in women of all races [1]. Roughly one out of eight women will develop invasive breast cancer during her life [2]. However, the mortality of breast cancer has decreased rapidly after 1989, with a total decline of 39% through 2015 [2]. This decline was attributed to improvements in early diagnosis and treatment [3]. Today, almost 90% of breast masses can be detected clinically or radiologically. However, more than 80% of surgically removed masses are eventually proved to be benign [4]. Therefore, the American College of Surgeons and the American Society of Breast Surgery recommend presurgical biopsy in all clinical and/or radiological masses. Surgical biopsy is the golden standard and provides a complete sampling of the radiologically identified lesions and their surroundings. However, the high incidence of the disease mandates percutaneous methods to be used in the majority of cases. Therefore, excisional breast biopsies were decreased and core-needle biopsies (CNB) were increased in number throughout the years [5]. However, the latter have the disadvantage of producing small samples that would cause inadequate assessment in up to a quarter of cases [6]. Vacuum-assisted biopsy (VAB) was recommended to overcome this obstacle. That technique allowed to obtain multiple cores from various locations, providing much larger but independent samples [7].

Over time, the size and thickness of the biopsy needles have increased from 14 to 16 G, enabling operators to perform lumpectomy during percutaneous biopsy. So called percutaneous lumpectomy, the latter has several theoretical advantages. Among them are the preservation of lesion architecture in the sample, a smaller residual tumor in the surgical bed, and a lower reresection rate in subsequent surgical treatment. Such advantages have resulted in the introduction of radiofrequency (RF)-assisted breast lesion excision systems (BLES) [8, 9]. These systems may deliver samples with intact architecture and clear margins, allowing better histological review. They may also reduce the disadvantages of open surgery such as hematoma, infection, and scar formation.

The purpose of the study was to evaluate the technical and diagnostic success and complications of BLES as a primary method of histopathological diagnosis and treatment of small and indeterminate breast masses.

## 2. Materials and Methods

### 2.1. Patients and Lesions

The study included a retrospective analysis of patients who were treated with BLES. The procedure was performed between 2014 and 2019 in the same center and by the same operator. During that period, 95 females with ages between 27 and 70 (50.5 ± 8.4) were treated, and one of them had two independent lesions. These patients were referred by surgeons to exclude a subtle focus of malignancy in BI-RADS 3 and 4a lesions and/or per subjective concerns. Thirty-two of these cases were sampled with CNB within the previous two months but were rereferred for indications that were stated previously. Inclusion criteria for the treatment were size (i.e., 20 mm or less in greatest diameter), depth (i.e., lesion-skin distance of 10 mm or more), and a BI-RADS classification of 3 or 4a. In the context of the latter criterion, 74 lesions were classified as BI-RADS 3 and 22 lesions were classified as BI-RADS 4a.

### 2.2. Interventional Procedure

**Figure 1:**
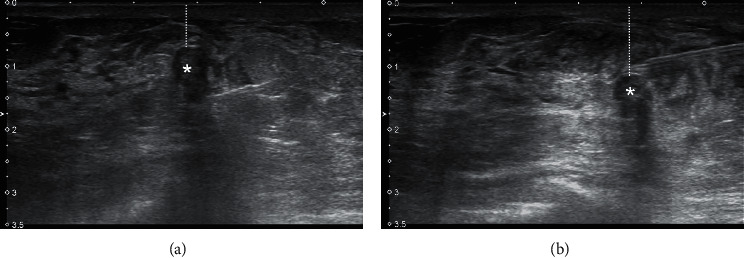
Expanding the lesion-to-skin distance (dotted lines) to prevent skin breech and burn. Large amount of prilocaine was injected around the lesion (*∗*) for hydrodissection and padding. (a) The distance was 6.4 mm. (b) The distance was augmented to 11.5 mm.

**Figure 2:**
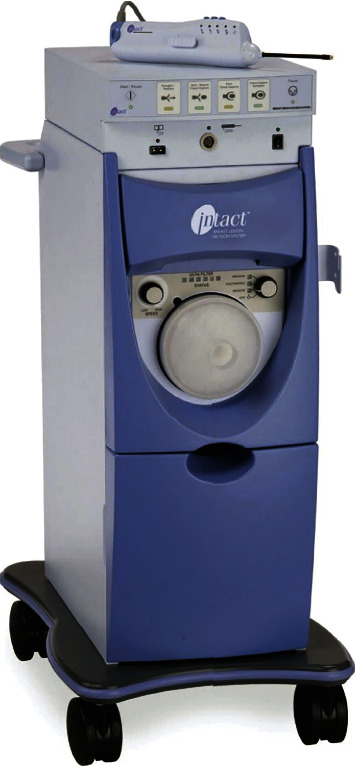
BLES system. The equipment consists of a base system with an RF generator and a vacuum evacuator, and a hand-held retriever handle with a removable probe.

**Figure 3:**
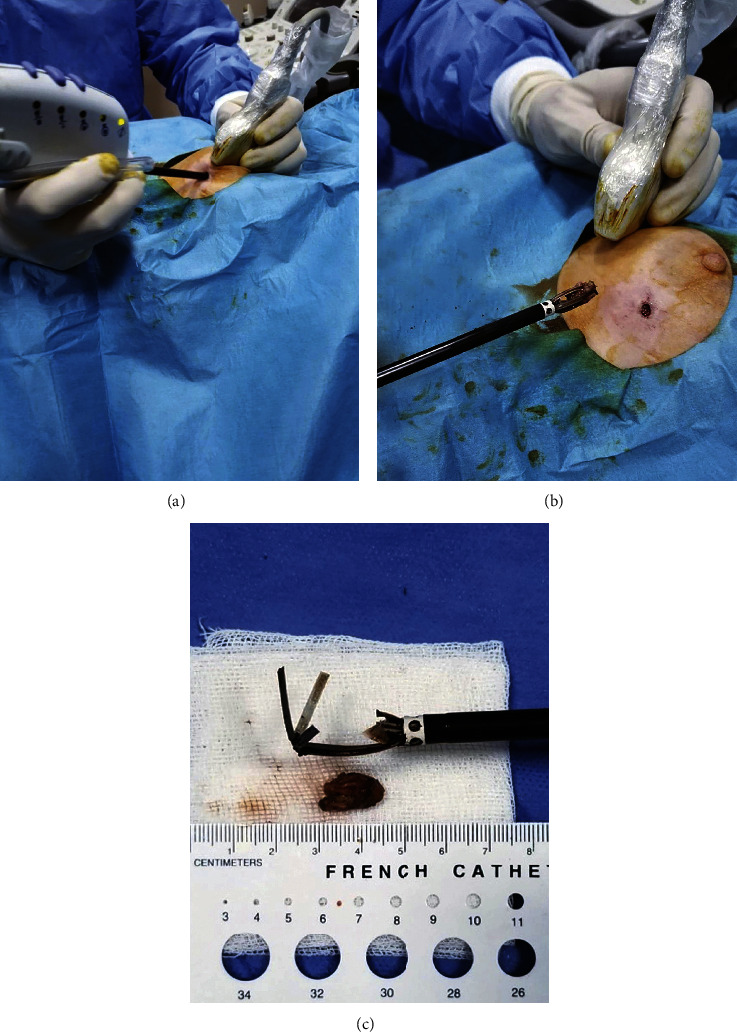
BLES probe. (a) The system allows the user to manage the entire process with one hand. The handle has several control buttons, identical to the ones that exist on the base system. This design minimizes the need for support technical personnel and allows the user's eye to be on the US screen during the procedure. (b) Withdrawal of the lesion within the basket. (c) Basket that was cut to free the excised mass.

**Figure 4:**
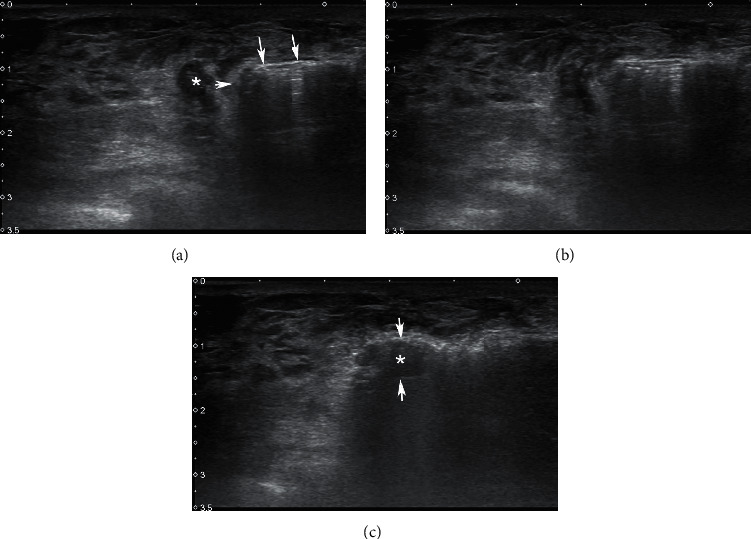
Removal of a neoplastic lesion (*∗*) with BLES. (a) Probe placement by the freehand method during light stabilization (long arrows, probe's upper edge; short arrow, probe's tip). (b) Probe tip was placed at the near end of the lesion. (c) Deployment of the basket (arrows). In this case, the lesion-to-skin distance was originally 8 mm and was expanded to 12 mm after the injection of prilocaine into the lesion's periphery.

The procedure was performed on an outpatient basis under ultrasound (US) guidance. 20 to 40 ml of prilocaine hydrochloride was employed to the approach path at the beginning of the procedure. It provided local anesthesia and was also used to subjectively evaluate the tissue density, to create a sleeve around the lesion and to improve the lesion to skin distance when needed (Figure 1). Lesions were excised using the BLES (formerly: Intact, Intact Medical Corporation, USA; recently: BLES, Medtronic Inc., Ireland) system (Figure 2). The basic component of the system was a vacuum-assisted retriever probe (also known as wand) that worked with RF energy (Figures 3(a)–3(c)). The rod-shaped probe was about 6.6 mm (approximately 6 G) × 11.4 cm in size. Different basket sizes were available, the smallest being 12 mm and the largest being 20 mm. These specified sizes represented upper limits that were always more than the actual basket lengths. We have used 12 mm baskets for lesions less than 8 mm in size, 15 mm baskets for lesions between 9 and 12 mm in size, and 20 mm baskets for lesions between 13 and 20 mm in size. The RF probe was advanced into the breast parenchyma through an incision that was made approximately 8 to 10 mm wide and 10 mm deep. RF probe placement was guided by US using the free-hand method during light stabilization of the breast with the help of the US probe (Figure 4(a)). The stabilization was applied with caution to cause minimal compression and to allow a sufficient gap to remain between the mass and the skin surface. The RF probe was placed at the near end of the long axis of the lesion, allowing its pointed tip to slightly push and penetrate the lesion for about 1 mm (Figure 4(b)). This practice was used to prevent the presence of normal parenchyma between the tip and the lesion that may otherwise cause excessive thermal generation or partial excision of the lesion.

**Figure 5:**
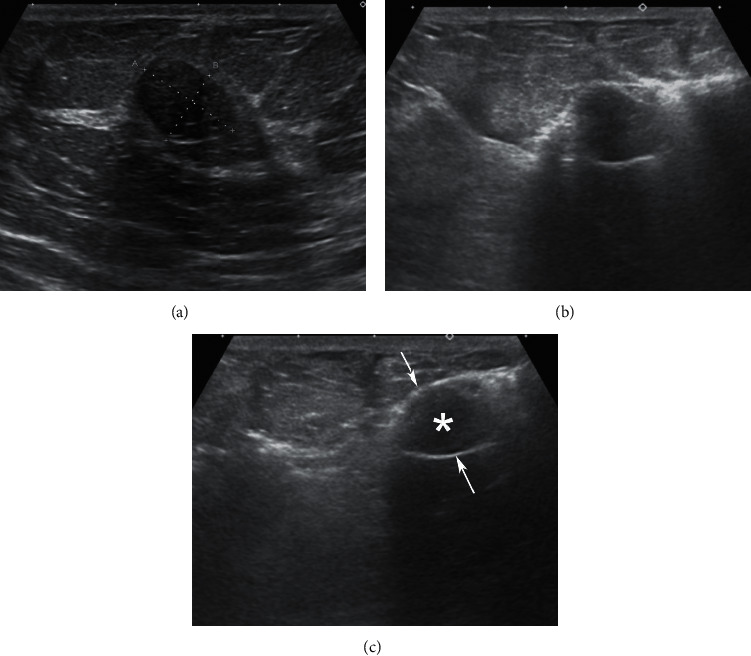
Removal of a benign lesion with BLES. (a) The mass. (b) Supporting elements of the basket during firing. (c) The mass (*∗*) was compressed and withdrawn in the cage (arrows).

Five small wires that formed an elastic circular RF ring were deployed from the probe to circumscribe the lesion after the firing (Figures 3(b), 4(c), 5(a), and 5(b)). The RF system automatically adjusted the energy level to the lowest possible level. It also evacuated gases and liquids that were collected at the tip of the RF probe for better performance. As the ring proceeded, it drew out four supporting elements which cradled the sample for en-block withdrawal (Figures 3(c), 4(c), and 5(c)). The procedure allowed a tissue with a diameter of 10 to 20 mm to be excised in less than ten seconds. A cold pack was applied to the excision site immediately before and after the firing to prevent bleeding and/or burning.

### 2.3. Follow-Up

All patients had a postprocedure follow-up within two weeks after the procedure to discuss pathology results, to examine the healing process of the incisions, and, if present, to deal with any complications such as hematoma or infection.

### 2.4. Pathological Evaluation

All relevant tissues were processed and paraffin embedded in cassettes from which serial sections were prepared. They were examined using standard pathological analysis (hematoxylin and eosin staining) as well as advanced pathological evaluation such as immunohistochemistry. Margin assessment was performed as described further in this study.

### 2.5. Data Extraction and Classification

Data were abstracted from pathology, diagnostic radiology, and interventional radiology reports. The technical success rate was defined as the percent relative frequency of excision of the target at the first attempt. The diagnostic success rate was defined as the percent relative frequency of sufficient samples for histopathological review. The complete excision rate was the percent relative frequency of so called negative margin (i.e., absence of microscopically confirmed disease at the margin in nonbenign (i.e., premalignant and malignant) lesions). Concordance between the histopathological diagnosis obtained with BLES and with previous CNB was assessed for cases for whom the latter was available by categorizing lesions under the three categories (such as benign, premalignant, and malignant). Complications were defined as skin burns, skin breech, bleeding/hematoma, and infection.

### 2.6. Statistical Analysis

Statistical evaluation was performed using IBM SPSS Statistics (version 27, IBM). The data were described using descriptive statistical methods. Occurrences were given in frequency (*n*) and in percentage (%) of all patients, as described in the previous subsection. Minimum (min), mean, maximum (max) values, and standard deviations (SD) were indicated as min-max (mean ± SD). The marginal homogeneity test was used to analyze the number of disagreements between BLES and CNB regarding paired proportions of categorized (i.e., benign, premalignant, and malignant) findings. *P* value was reported in an opened form, and *P* < 0.05 was chosen as the level of significance.

### 2.7. Research Ethics Standards Approval

The study was approved by the Institutional Review Board (approval no: 25-05-2021/5). This was a retrospective study, and informed consent was obtained for the procedures.

## 3. Results

Lesions were between 5 and 20 (12.3 ± 3.8) mm in length. 12 mm baskets were used in 16, 15 mm baskets in 28, and 20 mm baskets in 52 lesions. Accordingly, the longest diameter of lesions was between 5 and 8.5 (7.1 ± 1.0) mm, 6.4 and 12 (9.9 ± 1.4) mm, and 12 and 20 (15.3 ± 2.3) mm for 12, 15, and 20 mm baskets, respectively.

In all but one patient, masses were removed at the first attempt. In a patient who had an extremely dense breast, the initial attempt ended with RF-ring deployment failure. In that case, the mass was removed using a second probe during the same session. Accordingly, the technical success rate was 98.95%. Of note was the presence of another case in which the lesion was excised but an empty basket was retrieved. In that case, the sample was found to be impacted under the skin and was manually removed using a pick-up during the same session.

**Figure 6:**
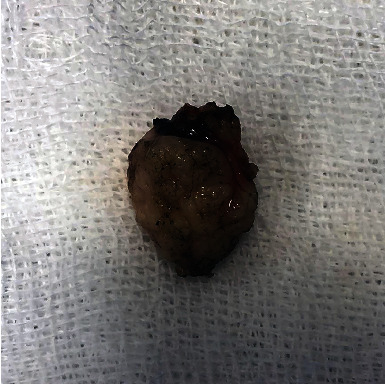
Gross examination of an excised mass. The diathermic effect (i.e., carbonization) was minimal and limited only to a few areas.

All samples were adequate for a confident histopathological diagnosis. Accordingly, the diagnostic success rate was 100%. The diathermic effect was found to be less than a millimeter (Figure 6). Ninety-one lesions were benign (60 fibroadenomas, 12 fibrosis/adenosis, 8 fibrocystic changes, 4 granulomatous mastitis, 5 intraductal papillomas, one lymphoid tissue, and one complicated cyst). Five lesions were neoplastic. Of them, two were atypical lobular hyperplasia, two were atypical ductal hyperplasia, and one was invasive ductal carcinoma. Surgical margins were found to be negative in two of these neoplastic cases. Accordingly, the complete excision rate, excluding benign lesions that were not analyzed for surgical margins, was 40%. The mean length for masses with complete excision was 12.1 mm, and the mean length for masses with incomplete excision was 12.8 mm.

When categorized as benign, premalignant, and malignant, there were 30 ties out of 31 cases which had both CNB and BLES. The difference between CNB and BLES regarding categorization was not significant (marginal homogeneity test, *P*=0.317). However, the number of discordant cases was much higher regarding subcategories, such as fibroadenoma vs. other benign pathologies. Of the five neoplastic lesions, only two had prior CNB. With that sampling method, one of them was diagnosed as a high-risk (atypical ductal hyperplasia) and the other as a benign (fibrocystic change) lesion. In that context, BLES upgraded one lesion (Table 1). Regarding BI-RADS classifications, two lesions that were previously classified as BI-RADS 3 were histopathologically diagnosed as neoplasia (atypical lobular hyperplasia), and nineteen lesions that were previously classified as BI-RADS 4a were histopathologically diagnosed as benign (Table 2).

**Figure 7:**
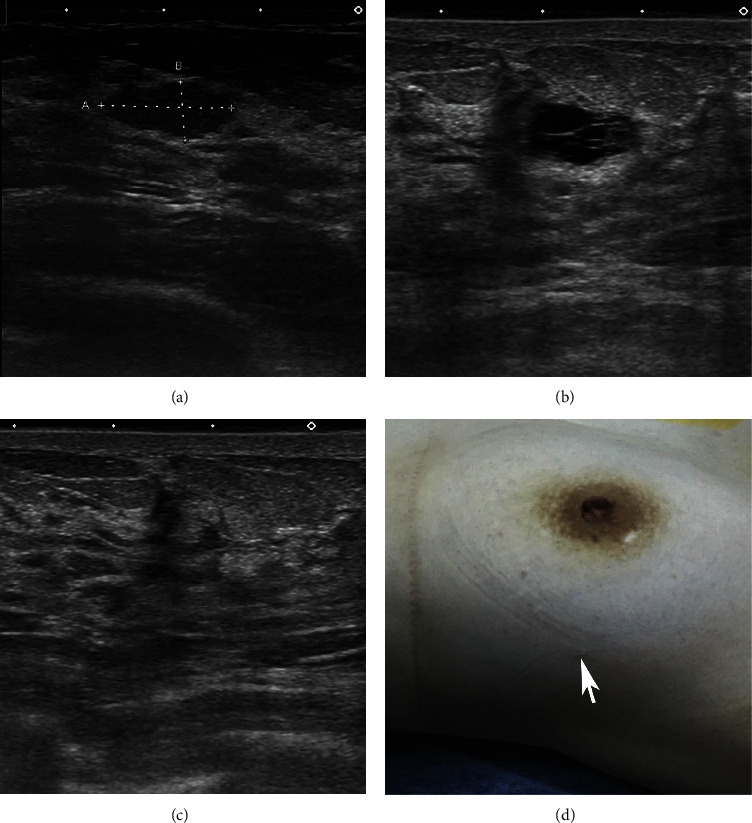
(a) Preoperative US image of a benign mass to be excised. (b) Self-limiting hematoma in the control scan. Its dimensions (12.5 × 6.5 mm) were approximately equal to the dimensions of the mass (13.5 × 6.0 mm) that has been removed. (c) Control US examination 2 weeks after simple aspiration. (d) Visual inspection of the BLES entry site (arrow) at sixth month.

No major complications were encountered during the procedure. There were no skin burns or breeches. The only minor complication was bleeding in two cases, presented as focal hematoma and treated with simple aspiration (Figures 7(a)–7(c)). Accordingly, the minor complication rate was 1.58%. No patients have developed distortion or collapse in the breast (Figure 7(d)). Patients were followed for 18 months or more during which no recurrence was observed.

## 4. Discussion

Preoperative diagnosis reduces unnecessary surgical excision of benign tumors. It also significantly reduces re-excision rates in breast cancer by predicting initial margin status in patients undergoing breast conservation [10]. Therefore, biopsy is recommended in all patients presenting with abnormal radiological findings. Although CNB is widely used in clinical practice, it does not always serve that purpose, mainly because it provides small samples. The materials obtained with CNB are barely 30 mg in weight [4]. VAB may provide larger samples; however, they are numerous and independent of each other [7].

BLES, on the other hand, may obtain a single intact sample up to 3 gr in weight. With such sampling, the technical success relative frequency of an undisrupted sample with preserved tissue architecture reported in the relevant literature was up to almost 100% [11]. In our study, the technical success rate was 98.95%. There was only one case where an empty basket was retrieved. According to the relevant literature, the relative frequency of such an event is 0.6–3.6% [12]. Such cases are usually due to the failure of the basket to deploy or to the presence of a very tight entry point or access path. According to our observations, meticulous hydrodissection may prevent the occurrence of the former (i.e., basket malfunction), but a second probe is required to complete the procedure if it occurs.

When the sampled abnormality is not histologically benign, tissue margins must be examined for completeness of removal. The resection zone in BLES is less than one millimeter with minimal diathermic (i.e., carbonization) effects that provide a confident assessment of border integrity (Figure 6). Complete excision rate of BLES is between 0 and 76% according to previous reports [11–14]. In this study, this rate was found to be 40%, although the number of malignant cases was too low to reach a solid conclusion regarding this parameter. The large variation in the literature regarding the rate of complete excision raises some questions about the use of BLES as a therapeutic device [12, 14]. However, it is not uncommon for patients to undergo more than one surgical procedure before achieving tumor-free margin even in surgery [15]. The necessity of the complete excision rate may be considered in that context. Nevertheless, the bulk of the lesions can be removed with BLES, hence providing breast conserving surgery with a smaller tumor even in cases with incomplete excision.

As abovementioned, BLES is superior to surgical excisional breast biopsy and VAB. Although VAB is a well-established alternative to surgical biopsy, previous studies demonstrated the supremacy of BLES over VAB [8, 9, 16]. In the former method, lesions are removed without being fragmented into small parts, so the architecture remains intact. Therefore, histopathological diagnosis can be made more accurately in suspicious lesions such as atypical ductal hyperplasia and ductal carcinoma in situ with lower underestimation rates [17]. In that context, the relative frequency for benign pathologies was 94.8%. This rate was lower (80%) in some earlier studies [8]. This difference was possibly caused by higher referral by clinicians to exclude a subtle focus of malignancy in possibly benign lesions in patients with known risk factors or due to patients' subjective concerns. These patients, except twenty-two, were classified as BI-RADS 3, and two of these BI-RADS 3 lesions were subsequently diagnosed neoplastic. One of these two neoplastic lesions (atypical lobular hyperplasia) had a previous CNB sampling that was reported as benign. The other had no previous CNB sampling. These frequencies were low to reach a conclusion on the accuracy of BLES. However, the technique has higher accuracy and a possibly lower rate of upgrade in surgery than CNB according to earlier studies [18, 19].

The complication rate for BLES was less than two percent and was comparable to other biopsy techniques, including VAB. These were minor bleedings and were self-limited. As the system was designed to emit RF energy to the surrounding tissue for synchronous excision and hemostasis (i.e., cauterization), it has a lower risk of bleeding compared to the latter. Nevertheless, the presence of bleeding diathesis and the use of antiplatelet and/or anticoagulant agents are still considered as relative contraindications. One may encounter additional complications including entry site burns, ablation zone skin burns in small breasts, and skin burns where grounding pads are located. These were not encountered in this study due to the meticulous use of precautionary measures as further detailed. Thermal burn at the entry site may be prevented by avoiding short tracts to the lesion. Thermal burn at the ablation site is prevented by allowing a safe distance between the lesion, the skin, and the thoracic wall. In that context, avoiding small breasts, empirical application of cold packs pre and postprocedurally, and administration of tumescent anesthesia to create a sleeve around the lesion are precautionary measures. Sleeves may help to increase the “lesion to skin” and the “lesion to the thoracic wall” distances and help to decrease the RF energy by facilitating excision (Figure 1). Grounding pad burns are prevented by establishing a firm surface contact between the entire pad and the skin and using adhesion tapes to reveal tension off the wires [20].

Apart from the performance measures and complications that were discussed above, BLES has certain advantages over excisional and percutaneous biopsy techniques and some disadvantages. It can be applied without sedation, in a way that causes the least discomfort to the patient and with minimal complications. The process can be carried out in less than 15 minutes in many cases, and in less than half an hour in all cases. However, it requires a considerable learning curve to perform. The operator must be extremely accurate and confident with the needle positioning, as once the RF-ring is deployed, no further adjustments can be made and the excision sequence cannot be terminated prematurely. Propagation of the wand in dense breasts is another problem. In such cases, exerting too much forward pressure may result in the sudden and uncontrollable advancement of the wand deep into the breast, and may damage the tissues next to the lesion. The skin incision required to introduce the BLES is significantly larger than the core and most vacuum needles. Nevertheless, the incision is only about 6–8 mm and can be managed with a single stitch and/or stripe. Cosmetic results are excellent, constituting an important reason for the system to be preferred over excisional biopsy by patients (Figure 7(d)).

In summary, BLES delivers a surgical quality specimen for a confident histopathological examination of breast masses. Acquisition of a greater amount of tissue and preservation of lesion architecture clearly constitute the advantage of BLES over other percutaneous biopsy techniques. Compared with surgical biopsy, BLES is at least as effective and safe and has all the other advantages of being a minimally invasive method. The method may provide a powerful alternative to surgical resection in suspicious lesions smaller than 20 mm since it enables the appropriate breast lesions to be effectively managed in an essentially one-stop outpatient procedure. In that context, it constitutes an effective and efficient example of minimally invasive therapeutic surgery allowing total removal of the lesion in many patients while preserving breast integrity and good cosmetic outcome in all patients.

## Figures and Tables

**Table 1:** Pairwise comparison of BLES and CNB results.

Tissue diagnosis	Sampling method	
	BLES (*n*)	CNB (*n*)
*BLES only* ^*∗*^		
Fibroadenoma	44	—
Papilloma	1	—
Premalignant	2	—
Malignant	1	—
Others (benign)	17	—
Total	65	

*Concordant cases*		
Fibroadenoma	7	7
Papilloma	1	1
Premalignant	1	1
Malignant	—	—
Others (benign)	9	9
Total	18	18

*Discordant cases*		
Fibroadenoma	9	Others (9)
Papilloma	3	Fibroadenoma (1)
		Others (2)
Premalignant	1	Other (1)
Malignant	—	—
Others (benign)	—	—
Total	13	13

CNB, core needle biopsy; BLES, breast lesion excision system; *N*^*∗*^, CNB was not performed.

**Table 2:** Initial BI-RADS classifications of benign and neoplastic lesions.

Histopathology	Radiology	
	BI-RADS 3	BI-RADS 4a
*Benign lesions*		
Fibroadenoma	50	10
Fibrosis/adenosis	7	5
Fibrocystic changes	8	—
Granulomatous mastitis	4	—
Intraductal papilloma	2	3
Lymphoid tissue	1	—
Complicated cyst	—	1

*Neoplastic lesions*		
Atypical lobular hyperplasia	2	—
Atypical ductal hyperplasia	—	2
Invasive ductal carcinoma	—	1
Total	91	5

BLES, breast lesion excision system.

## Data Availability

The data used to support the findings of this study are restricted by the Institutional Review Board (Fatih Sultan Mehmet Training and Education Hospital) in order to protect patient privacy. Anonymized data used to support the findings of this study are available from the corresponding author upon request.
